# Supplementary material for: Heat Exposure among Adult Women in Rural Tamil Nadu, India
Source: Environ Sci Technol. 2023 Dec 28;58(1):315–22. doi: 10.1021/acs.est.3c03461 (PMC10785747; doi:10.1021/acs.est.3c03461)
Supplement: Supplementary file 1 — es3c03461_si_001.pdf [file es3c03461_si_001.pdf]

SUPPORTING INFORMATION

Heat Exposure Among Adult Women in Rural Tamil Nadu, India

Aniruddha Deshpande[1], Noah Scovronick[2], Thomas F. Clasen[2], Lance Waller[3], Jiantong Wang[3], Vigneswari Aravindalochanan[4], Kalpana Balakrishnan[4], Naveen Puttaswamy[4], Jennifer Peel[5], Ajay Pillarisetti[6]

[1] Department of Epidemiology, Rollins School of Public Health, Emory University, Atlanta, GA, USA 30322

[2] Gangarosa Department of Environmental Health, Rollins School of Public Health, Emory University, Atlanta, GA, USA 30322

[3] Department of Biostatistics and Bioinformatics, Rollins School of Public Health, Emory University, Atlanta, GA, USA 30322

[4] Sri Ramachandra Institute of Higher Education and Research, Chennai, India

[5] Department of Epidemiology, Colorado State University, Aurora, CO, USA 80523

[6] Division of Environmental Health Sciences, University of California Berkeley, Berkeley, CA, USA 94720

Total number of pages: 3

Cover page (page S1)

Supporting Figure S1. Scatterplots and simple correlations comparing personal exposures with ambient monitors and modelled products on extreme heat days, defined as days where the nearest GHCN monitor registered a maximum temperature > 35 °C. (page S2)

Supporting Figure S2. Bland-Altman plots comparing personal exposures with ambient monitors and modelled products on extreme heat days, defined as days where the nearest GHCN monitor registered a maximum temperature > 35 °C. (page S3)

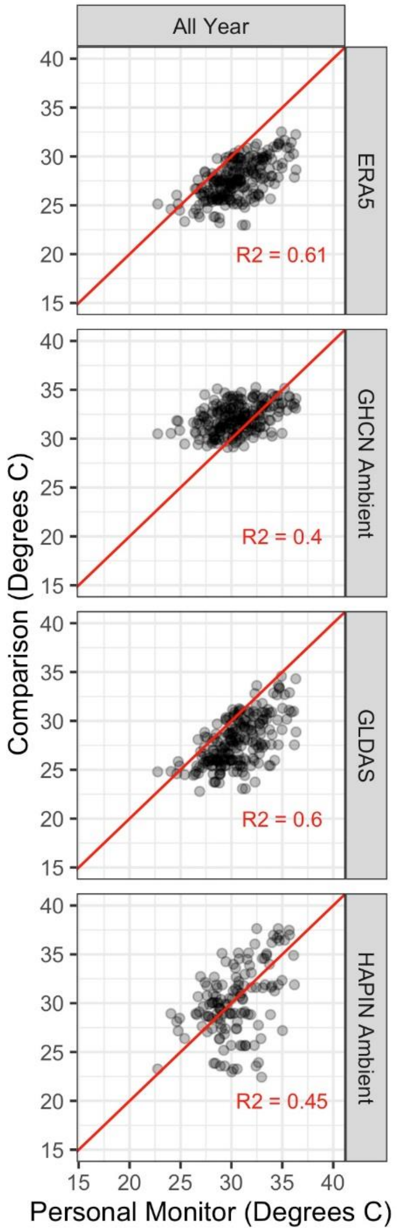

Supporting Figure S1: Scatterplots and simple correlations comparing personal exposures with ambient monitors and modelled products on extreme heat days, defined as days where the nearest GHCN monitor registered a maximum temperature > 35 °C. Red lines are 1:1 lines; points represent daily average values.

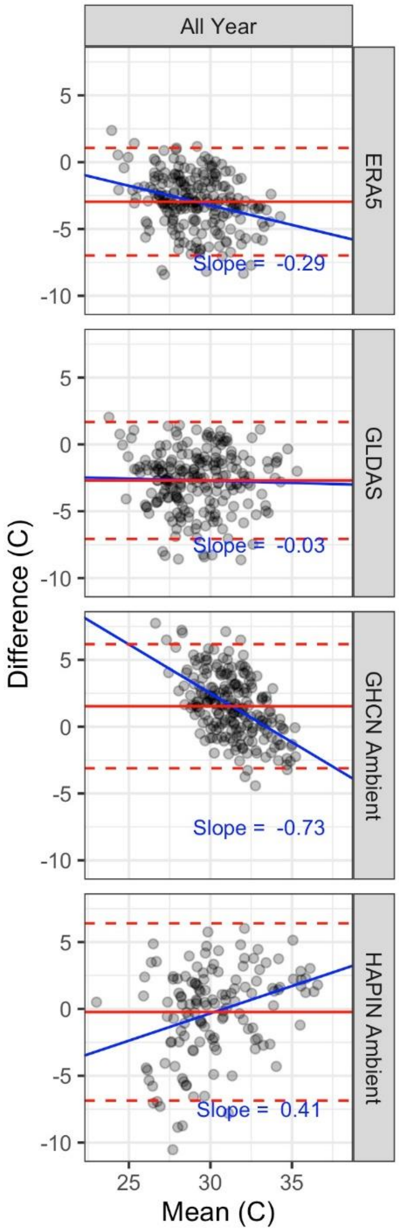

Supporting Figure S2. Bland-Altman plots comparing personal exposures with ambient monitors and modelled products on extreme heat days, defined as days where the nearest GHCN monitor registered a maximum temperature > 35 °C. Blue sold lines are best-fit regression lines displaying the relationship between bias and mean changes in daily temperature. Dashed red lines are 95% Wald confidence intervals; solid red lines are mean values.
